# Host Plant Trichome Architecture Mediates the Predatory Efficacies of *Nesidiocoris tenuis* and *Chrysoperla carnea* Against *Tuta absoluta*

**DOI:** 10.3390/insects17090974

**Published:** 2026-09-21

**Authors:** Juhyeok Lee, Hwal-Su Hwang, Lisheng Zhang, Yuyan Li, Kyeong-Yeoll Lee

**Affiliations:** 1 Department of Plant Medicine, College of Agriculture and Life Sciences, Kyungpook National University, Daegu 41566, Republic of Korea; dong3272@naver.com (J.L.); bgtwo2@naver.com (H.-S.H.); 2 State Key Laboratory for Biology of Plant Diseases and Insect Pests, Institute of Plant Protection, Chinese Academy of Agricultural Sciences, Beijing 100193, China; zhangleesheng@163.com; 3 Institute of Plant Medicine, Kyungpook National University, Daegu 41566, Republic of Korea

**Keywords:** *Tuta absoluta*, *Nesidiocoris tenuis*, *Chrysoperla carnea*, glandular trichomes, biological control

## Abstract

The tomato leafminer, *Tuta absoluta*, is a globally important pest that causes severe losses in tomato production, and effective biological control strategies are increasingly needed due to widespread insecticide resistance. The effectiveness of natural enemies is influenced by plant structural traits, such as trichomes, which create complex microhabitats that shape predator–prey interactions. Here, we compared the predatory performances, plant-level control efficacies, and predator–plant interactions of two widely used biological control agents, *Nesidiocoris tenuis* and *Chrysoperla carnea*, against *T. absoluta* eggs on tomato and eggplant. In laboratory assays, predation capacity increased with predator developmental stage, with adult female *N. tenuis* and third-instar larvae of *C. carnea* exhibiting the highest species-specific predation rates. However, in whole-plant and semi-enclosed cage experiments, *N. tenuis* maintained substantial foraging activity, effectively suppressing *T. absoluta* on tomato plants, whereas *C. carnea* showed limited control efficacy, with 86.7% of larvae abandoning plants within 24 h. Conversely, *C. carnea* exhibited significantly higher predation rates than *N. tenuis* on eggplant, with minimal abandonment. Comparing plant surface architectures revealed that tomato leaves possessed substantially longer trichomes than eggplant leaves (4.14 vs. 0.48 mm), which restricted the foraging activity of *C. carnea*. Our results indicated that *N. tenuis* remains the preferable agent for tomato, while *C. carnea* is highly suitable for short-trichome-bearing solanaceous crops like eggplant. Crop surface characteristics strongly influence biological control effectiveness, and matching predator traits with crop architecture will improve the success of natural enemy-based pest management.

## Simple Summary

The tomato leafminer (*Tuta absoluta*) is a serious pest that threatens tomato production worldwide, and sustainable alternatives to chemical control are urgently needed. Biological control using natural enemies is an important strategy, but the success of predators may depend on the characteristics of the crops on which they are released. In this study, we compared the abilities of two important predators, *Nesidiocoris tenuis* and *Chrysoperla carnea*, to control *T. absoluta* eggs on tomato and eggplant plants. Although both predators showed strong predatory ability under laboratory conditions, their efficacies differed greatly on different crops. While *N. tenuis* provided robust control on tomato plants, *C. carnea* showed limited control because the long hairs on tomato leaves interfered with its movement and hunting behavior. In contrast, *C. carnea* was highly effective on eggplant, which has shorter leaf hairs that allow easier movement. These results demonstrate that plant characteristics can strongly influence the success of biological control and highlight the importance of selecting natural enemies that are well adapted to specific crops.

## 1. Introduction

The tomato leafminer, *Tuta absoluta* (Meyrick) (Lepidoptera: Gelechiidae), is recognized as one of the most destructive pests affecting tomato cultivation worldwide [1]. Larvae feed within the mesophyll layer of leaves, forming characteristic galleries and directly damaging plant tissues. Without proper management, yield losses can reach as high as 80–100% [2]. Though native to South America, *Tuta absoluta* was originally described based on specimens from Huancayo, in the central highlands of Peru. It was first reported in Spain in 2006 and, since then, has rapidly spread throughout Europe, Africa, and Asia. In 2024, it was detected in Korea [3,4,5,6].

The control of *T. absoluta* has relied primarily on chemical insecticides; however, the development of insecticide resistance, along with the negative impacts of pesticides on non-target and sometimes beneficial organisms, has created a need for alternative pest management strategies. Biological control has emerged as a vital strategy [7,8,9]. While the term “biological control” encompasses multiple strategies, including conservation and classical biological control, this study focuses on applied (augmentation) biological control, in which mass-reared natural enemies are directly released into agroecosystems to suppress pest populations [10].

Among the primary natural predators of *T. absoluta*, the mirid bug *Nesidiocoris tenuis* (Reuter) (Hemiptera: Miridae) is widely used due to its high predatory capacity, with adults consuming up to 50–60 eggs daily [11]. However, as a zoophytophagous predator, *N. tenuis* can shift toward phytophagy when prey becomes scarce or predator densities are high, consuming plant sap to meet its nutritional and moisture requirements, which induces plant damage, including necrotic rings on stems and petioles and flower abortion, with subsequent yield loss [12,13]. Given these drawbacks, additional or complementary biological control agents should be explored. The green lacewing, *Chrysoperla carnea* (Stephens) (Neuroptera: Chrysopidae), is a highly promising candidate. Its third-instar larvae exhibit exceptional voracity, consuming up to 36 ± 2 *T. absoluta* eggs per day under ad libitum laboratory conditions [14], but while transitioning into adulthood, the mouthparts of *C. carnea* undergo structural changes, which is accompanied by a dietary shift, and adults feed exclusively on nectar, pollen, and honeydew [15,16]. However, while the third-instar larvae’s baseline predation capacities in artificial arenas are well documented, comparative evaluations on intact host plants remain scarce.

The success of predation is often mediated by the host plant’s architecture and surface structures, which dictate the physical environment in which predator–prey interactions occur [17]. Defensive plant structures, particularly trichomes, play a pivotal role in mediating plant–herbivore–predator interactions [18,19]. While dense or glandular trichomes serve as barriers to many generalist insects, *T. absoluta* utilizes the volatile organic compounds emitted by glandular trichomes as chemical cues for identifying suitable host species [20]. During oviposition, adult female moths circumvent physical surface defenses by placing eggs in protected microhabitats, such as leaf crevices or abaxial surfaces, where trichome interference is reduced [21]. Upon hatching, neonate larvae rapidly bore into the mesophyll to mine internally, effectively escaping surface trichomes [1]. Understanding such trait-mediated predator–plant interactions is vital to moving biological control evaluations beyond descriptive laboratory assays and toward hypothesis-driven, plant-level predictions.

This study evaluates the biological control efficacies of *N. tenuis* and *C. carnea* against *T. absoluta*, focusing on the mechanisms by which crop surface structure mediates *T. absoluta* egg predation. In addition to directly comparing prey consumption in an artificial setting, we conducted paired assays using two plant species, tomato, *Solanum lycopersicum* L. (Solanales: Solanaceae), and eggplant, *S. melongena* L. (Solanales: Solanaceae), as substrates and coupled the results with trichome characteristic assessments. We hypothesized that (1) differences in epidermal trichome architecture (density and length) act as key physical barriers that differentially affect the foraging performances of *N. tenuis* and *C. carnea* and (2) *N. tenuis* possesses specialized biomechanical and behavioral adaptations that allow it to overcome the barriers posed by dense trichomes more effectively than *C. carnea*. Our findings elucidate how crop surface variations mediate predator efficacy, offering actionable insights for optimizing integrated pest management (IPM) in greenhouse systems.

## 2. Materials and Methods

### 2.1. Plants

Two economically important solanaceous host plants with contrasting surface structures were utilized to isolate the effects of plant architecture on predator performance: tomato (*S. lycopersicum* cv. ‘Seogwang’; Farm Hannong, Seoul, Republic of Korea) and eggplant (*S. melongena* cv. ‘Danong’; Danong Co., Dongducheon, Republic of Korea). These specific cultivars are standard commercial varieties widely grown in East Asian greenhouse systems, ensuring the study’s agronomic relevance, and exhibit highly divergent, visually distinct trichome phenotypes (long and sparse vs. short and dense).

Seeds of both species were sown individually in 9 cm × 9 cm × 8 cm plastic pots filled with a standardized 1:1 (*v*/*v*) substrate mixture of commercial nursery and culture soils. To ensure physiological and morphological uniformity across replicates, seedlings were selected for experiments once they reached a vegetative stage characterized by 4–5 fully expanded true leaves and an average height of 15–20 cm (after approximately two weeks of initial growth). All plants were cultivated in an environmental growth chamber under identical, strictly controlled conditions: 25 ± 1 °C and 70 ± 5% relative humidity (RH), with a 16:8 h light–dark photoperiod, using cool white fluorescent lighting to prevent any light- or temperature-induced variations in trichome ontogeny.

### 2.2. Pest and Predators

The *T. absoluta* colony originated from infested material provided by the Gyeonggi-do Agricultural Research and Extension Services, Hwasung, Republic of Korea, in 2024. The colony was maintained on healthy 65–75 cm tall tomato plants (cv. ‘Seogwang’) following standard rearing methods for *T. absoluta* [1]. Cages were checked daily, and host plants were replaced with fresh individuals when larval foliar consumption reached approximately 80%, maintaining stable, uncrowded rearing conditions.

*Nesidiocoris tenuis* adults were purchased from the Insect Industry Research Institute (Nonsan, Republic of Korea). Adults and nymphs were reared on potted tobacco plants, *Nicotiana tabacum* L. (Solanales: Solanaceae), which provided the oviposition substrate, and fed mass-reared eggs of the almond moth, *Cadra cautella* (Walker) (Lepidoptera: Pyralidae), as a factitious food source, following established mirid bug rearing protocols [22].

*Chrysoperla carnea* was sourced as first-instar nymphs from Koppert Korea (Gwangju, Republic of Korea) and maintained for at least two generations to ensure acclimation to laboratory conditions before testing. Larvae were reared individually in multi-well units to prevent cannibalism and fed *C. cautella* eggs ad libitum, while adults were maintained in mesh cages and provided a yeast, honey, and water mixture (1:1:1 ratio) as a liquid diet, according to standardized lacewing rearing procedures [14,15].

All insect rearing and subsequent experimental assays were performed under the same environmental conditions: 25 ± 1 °C and 70 ± 5% RH, with a 16:8 h (L:D) photoperiod.

### 2.3. Artificial Predation Assays

Assays were designed around the distinct life-history traits of each predator. Since *N. tenuis* exhibits predatory behavior during both the nymphal and adult stages, its consumption was assessed across all developmental cohorts (the first five nymph stages [N1–N5], adult males, and adult females). Adult males and females were evaluated separately because females require substantially higher protein and energy intake to support oogenesis and continuous egg production, while males’ energy demands are primarily for somatic maintenance. On the other hand, as *C. carnea* adults are non-predatory [23], field releases strictly included larval-stage individuals, and evaluations for this species were restricted to larvae of the first three instars (L1–L3).

To standardize test cohorts, N1 *N. tenuis* nymphs were isolated immediately after emergence from oviposited eggs on tobacco leaves and reared individually in 50 mm × 15 mm breeding dishes until reaching the target stage. For *C. carnea*, larvae and eggs were monitored daily, and upon molting/hatching, L1, L2, and L3 individuals were held for 24–48 h to allow complete maturation prior to the assay to ensure physiological uniformity. Similarly, *N. tenuis* adults were used 3–5 days after emergence. All test insects were starved for 24 h in a humidified arena before the assay to standardize hunger levels and reduce variation in foraging behavior.

The target prey, *T. absoluta* eggs, was obtained using custom oviposition cages consisting of a 7.2 cm × 7.2 cm × 10 cm transparent polypropylene container (Kolabshop, Seoul, Republic of Korea) equipped with a water-filled 1.5 mL microtube used to maintain fresh tomato leaf material. Approximately 100 *T. absoluta* adults were introduced per cage and allowed to oviposit freely for 24–36 h. The egg density per experimental arena was then standardized to 350 eggs, with excess eggs carefully culled under a stereomicroscope (Fisher Scientific, Pittsburgh, PA, USA) using a fine, moistened camel-hair brush to avoid disturbing the remaining eggs. This egg density was selected based on pre-trials indicating it exceeded the maximum daily feeding capacity of the most voracious life stage (L3 *C. carnea* larvae), ensuring ad libitum access and that consumption rates were driven strictly by predator feeding capacity, never prey limitation.

Individual predators were introduced singly into 120 mm diameter × 80 mm tall breeding dishes containing a tomato leaf bearing the 350 eggs. Predation was quantified across two consecutive 24 h intervals (respectively spanning hours 0–24 and hours 24–48) to evaluate short-term voracity versus sustained feeding rates. At the 24 h mark, the predator was carefully guided onto a fresh, identically prepared leaf substrate bearing 350 new eggs, using a single-hair brush to minimize physical stress, and the initial leaf was removed for counting.

Predation was calculated as the difference between the initial egg count (350) and the remaining intact egg count. This metric effectively harmonized measurements between the species: while *N. tenuis* punctures eggs, leaving behind empty chorions, *C. carnea* frequently masticates or removes eggs entirely from the leaf surface. The artificial predation experiment was analyzed as a completely randomized design (CRD). Each experimental unit consisted of a single 120 mm diameter × 80 mm tall breeding dish (SPL, Seoul, Republic of Korea) containing one tomato leaf with 350 *T. absoluta* eggs (along with the replacement leaf) and an individual predator. Each treatment combination was evaluated across 15 independent replicates (*n* = 15).

### 2.4. Tomato Plant Predation Assays

To assess biological control efficacy in a more realistic environment with complex microhabitats, we evaluated the *T. absoluta* egg foraging capacities of adult *N. tenuis* females and third-instar *C. carnea* larvae on intact, whole tomato plants. These whole-plant experiments were conducted inside 24 cm × 24 cm × 50 cm polyester mesh cages containing a single vegetative-stage, 40–45 cm tall tomato plant.

The life stages selected for this assay represented the peak predatory cohorts identified in the artificial predation trials: adult *N. tenuis* females (4–5 days post-emergence) and third-instar *C. carnea* larvae (24–48 h post-molt). To standardize hunger levels and stimulate foraging activity, all tested individuals were starved for 24 h before release, as described in Section 2.3.

To establish a natural prey distribution across the plant canopy, a minimum of 100 *T. absoluta* adults were released into each plant-containing cage and allowed to oviposit freely for 24–36 h. After removing the adults, eggs were counted and systematically mapped and recorded across all leaflets, petioles, and stems using a portable digital microscope (Siwon Optical Technology, Anyang, Republic of Korea) and a 10× magnification hand loupe (Hyoryong Company, Seoul, Republic of Korea). The egg density per plant was then standardized as close to 350 eggs as possible, with excess eggs carefully culled using a fine, moistened camel-hair brush. Given the geometric complexity of intact foliage, final counts were verified independently by two observers, limiting spatial counting errors to <3% across all replicates.

A single predator (either an adult female *N. tenuis* or a third-instar *C. carnea* larva) was gently introduced onto the lowest true leaf node of the plant, using a soft brush to minimize handling stress and prevent immediate escape behavior. Each predator was allowed to forage undisturbed for 24 h, after which it was removed, and the plant was systematically disassembled. Stems and leaves were dissected into small segments, and the remaining intact eggs were counted under a high-resolution stereomicroscope. Total predation was calculated as the difference between the verified initial egg count and the residual egg count. The whole-tomato plant experiment was analyzed as a CRD. Each experimental unit consisted of an individual 24 cm × 24 cm × 50 cm polyester mesh cage containing a single vegetative tomato plant with 350 *T. absoluta* eggs and one predator. A total of 15 independent replicates were evaluated per treatment (*n* = 15).

### 2.5. Semi-Enclosed Cage Predation Assays

To evaluate the long-term suppression of *T. absoluta* on whole plants under realistic population dynamics, experiments were conducted in 55 cm × 50 cm × 80 cm polyester mesh cages containing a single 70–75 cm tall tomato plant. A pair of healthy adults, defined as actively flying individuals with intact appendages (wings, legs, and antennae) and no visible anatomical deformities, were held for three days to ensure mating and peak oviposition capacity before releasing five mated pairs per cage to oviposit freely. The adults were synchronized by rearing pupae in breeding dishes until eclosion, and one male and one female were paired within two days of emergence.

To simulate continuous field pest pressure, adult *T. absoluta* remained inside the cages throughout the experiment. Exactly twenty-four hours after pest introduction, predators were released, creating three distinct treatments: five adult *N. tenuis* females (within five days of eclosion), five third-instar *C. carnea* larvae (within two days of molting), or a predator-free control to enumerate baseline egg accumulation. Canopy-wide initial egg counts were recorded immediately before predator release (hour 0) using a portable digital microscope (Siwon Optical Technology) and a 10× hand loupe, with unconsumed eggs quantified using the same non-destructive methods 24 and 48 h post-release. Each treatment was replicated 15 times (*n* = 15) using fresh plants and insect cohorts.

### 2.6. Eggplant Predation Assays

To explicitly test whether trichome architecture acts as a primary physical barrier to predator foraging, whole-plant assays were conducted on eggplant, an alternative host featuring high densities of short, non-glandular trichomes [24]. This experiment was analyzed as a CRD, with each experimental unit consisting of an individual 24 cm × 24 cm × 50 cm polyester mesh cage, each housing a single 40–45 cm tall, vegetative-stage eggplant.

Age-standardized predators (adult 3–5-day-post-emergence *N. tenuis* females and 24–48 h post-molt third-instar *C. carnea* larvae) were used, matching the physiological profiles from the tomato assays. Due to lower oviposition preference on eggplant, natural oviposition (achieved using 100 *T. absoluta* adults for 24–36 h) was supplemented with fresh eggs, transferred using a fine brush under a stereomicroscope (Fisher Scientific), to establish a standardized density of exactly 350 eggs per plant. To prevent the use of eggs with damaged chorions and ensure natural adhesion, the structural integrity and physical stability of transferred eggs on the leaf surface were verified prior to predator introduction.

For each plant, a single predator was introduced and allowed to forage undisturbed for 24 h. Then, predators were removed, and residual eggs were counted under a stereomicroscope to determine total consumption (350 minus remaining eggs). Each treatment combination was evaluated across 15 independent replicates (*n* = 15).

### 2.7. Residency Behavior of Chrysoperla carnea and the Trichome Characteristics of Host Plants

To quantify the behavioral mechanism driving differences in control efficacy, the spatial residency of *C. carnea* third-instar larvae was recorded at the end of the 24 h exposure period of the whole-plant assays for both tomato and eggplant (*n* = 15 cages per plant species). Each larva was classified as “resident,” if actively attached to or foraging on any vegetative structure of the host plant (leaves, petioles, or stems), or “dislodged/departed,” if occupying the cage floor or mesh walls, completely abandoning the foliage. Larvae found on the container floor were inspected under a hand loupe (Hyoryong Company) to confirm survival and rule out mortality-induced dislodgement.

Following these behavioral observations, a quantitative micro-morphological analysis of host plant surface structure was conducted to correlate predator retention with specific architectural traits. Fifteen 70–80 cm tall vegetative plants per species were examined, with all surface measurements performed strictly in a 10 cm vertical zone on the main stem spanning 40–50 cm above the soil to eliminate variations caused by plant ontogeny or vertical stratification.

Trichome density was quantified by counting all visible trichomes within a linear 1 cm segment of the stem surface within the predefined zone under 20× magnification (Keyence Corp., Osaka, Japan). Trichome length, from the base on the epidermal surface to the tip, was determined by randomly measuring 30 distinct trichomes per plant within the same stem zone. Additionally, trichomes were categorized as glandular (exhibiting a secretory head) or non-glandular based on their structural morphology. Imaging and micro-measurements were performed using a VHX-7000N digital microscope with a VHX-E100 lens (Keyence Corp., Osaka, Japan) at 20–100× magnification, employing scale-calibrated digital imaging software Ver 1.01 (Keyence Corp.) to ensure sub-millimeter precision.

### 2.8. Data Analysis

All statistical analyses were performed using R software version 4.3.2 (R Foundation for Statistical Computing, Vienna, Austria). Prior to formal hypothesis testing, parametric assumptions, including the normality of residuals and homoscedasticity of variance, were evaluated using Shapiro–Wilk tests and Levene’s tests, respectively, with significance thresholds of *α* = 0.05. In instances where these assumptions were violated, Welch’s analysis of variance (ANOVA) and the non-parametric Kruskal–Wallis test were applied concurrently as a conservative validation step: when both approaches indicated statistical significance (*p* < 0.05), the primary parametric outputs are reported for consistency.

To evaluate artificial predation assay data, differences in consumption across developmental stages and between the two sequential feeding periods (hours 0–24 vs. hours 24–48) were analyzed using two-way ANOVAs based on Type III sums of squares to account for potential interactions between factors. When significant main effects or interactions were detected, pairwise differences were tested using Tukey’s honestly significant difference (HSD) post hoc tests (*α* = 0.05). For the single-factor whole-plant experiments (tomato and eggplant were analyzed separately), differences in mean egg consumption between the two predator species were analyzed using independent *t*-tests with significance thresholds of *p* < 0.05.

For the semi-enclosed cage assay simulating continuous population dynamics, treatment effects were analyzed by comparing the absolute numbers of *T. absoluta* eggs recorded at the final, 48 h endpoint among the three treatment groups using a one-way ANOVA followed by pairwise comparisons through Tukey’s HSD test (*p* < 0.05). The behavioral residency of *C. carnea* was presented as the proportion of individuals dislodged from the plant and statistically analyzed as a binomial categorical variable using Pearson’s chi-square (*χ*^2^) test of independence. Fisher’s exact test was performed simultaneously as a conservative verification for small sample sizes. Lastly, differences in trichome density and length between tomato and eggplant plants were analyzed using independent-sample *t*-tests (*p* < 0.05), applying Welch’s *t*-statistic and degrees of freedom corrections (i.e., Welch’s unequal variances *t*-test) when the assumption of homoscedasticity was violated.

## 3. Results

### 3.1. Predation in the Artificial Assay

**Figure 1:**
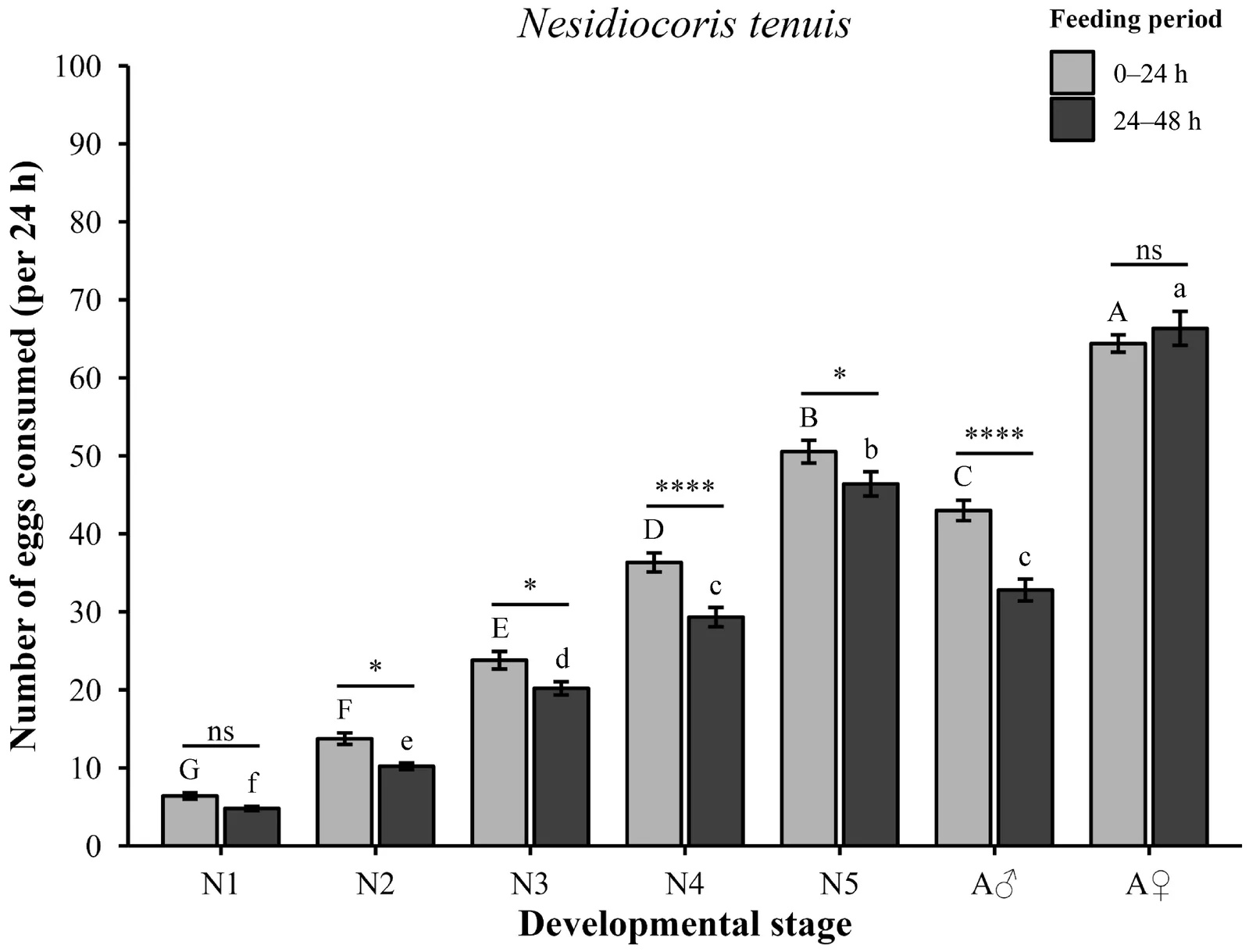
Predation capacity of *Nesidiocoris tenuis* in the artificial predation assay, presented as the mean (±SE; *n* = 15 replicates) number of *Tuta absoluta* eggs consumed by first- to fifth-instar nymphs (N1–N5), adult males (A♂), and adult females (A♀) across two sequential 24 h feeding periods respectively spanning hours 0–24 (light-gray bars) and hours 24–48 h (dark-gray bars) in a Petri dish arena. Different letters indicate significant differences between the means of different developmental stages, with uppercase letters used for the first feeding period and lowercase letters for the second (Type III ANOVA, Tukey’s HSD test, *α* = 0.05). Statistically significant differences between feeding periods at the same developmental age are denoted using asterisks (*, *p* < 0.05; ****, *p* < 0.0001), with “ns” denoting no significant difference.

For *N. tenuis*, a two-way Type III ANOVA revealed a highly significant interaction between predator developmental stage and feeding interval (*F*_6,196_ = 5.13, *p* < 0.0001). Regarding main effects, predation capacity varied significantly among developmental stages (*F*_6,196_ = 295.66, *p* < 0.001), whereas the effect of the feeding interval alone was not statistically significant (*F*_1,196_ = 1.29, *p* = 0.257) (Figure 1).

Tukey’s HSD post hoc comparisons revealed that predation capacity was strongly stage-dependent, increasing with nymphal development, and adult females exhibited the highest overall consumption across both time intervals (*p* < 0.001). Additionally, clear sex-based dimorphism was observed, as markedly lower feeding activity was recorded for adult males than for both adult females and N5 nymphs at the 24 h (*p* = 0.0003) and 48 h (*p* < 0.0001) timepoints. Regarding temporal foraging trends, a significant reduction in egg consumption between first (hours 0–24) and second (hours 24–48) intervals occurred across mid-to-late nymphal stages (N2: *p* = 0.0389; N3: *p* = 0.0354; N4: *p* = 0.0001; N5: *p* = 0.0159), but this temporal effect was not statistically significant for N1 nymphs (*p* = 0.348). Additionally, adult males exhibited sharp decreases in predation over time (*p* < 0.0001).

**Figure 2:**
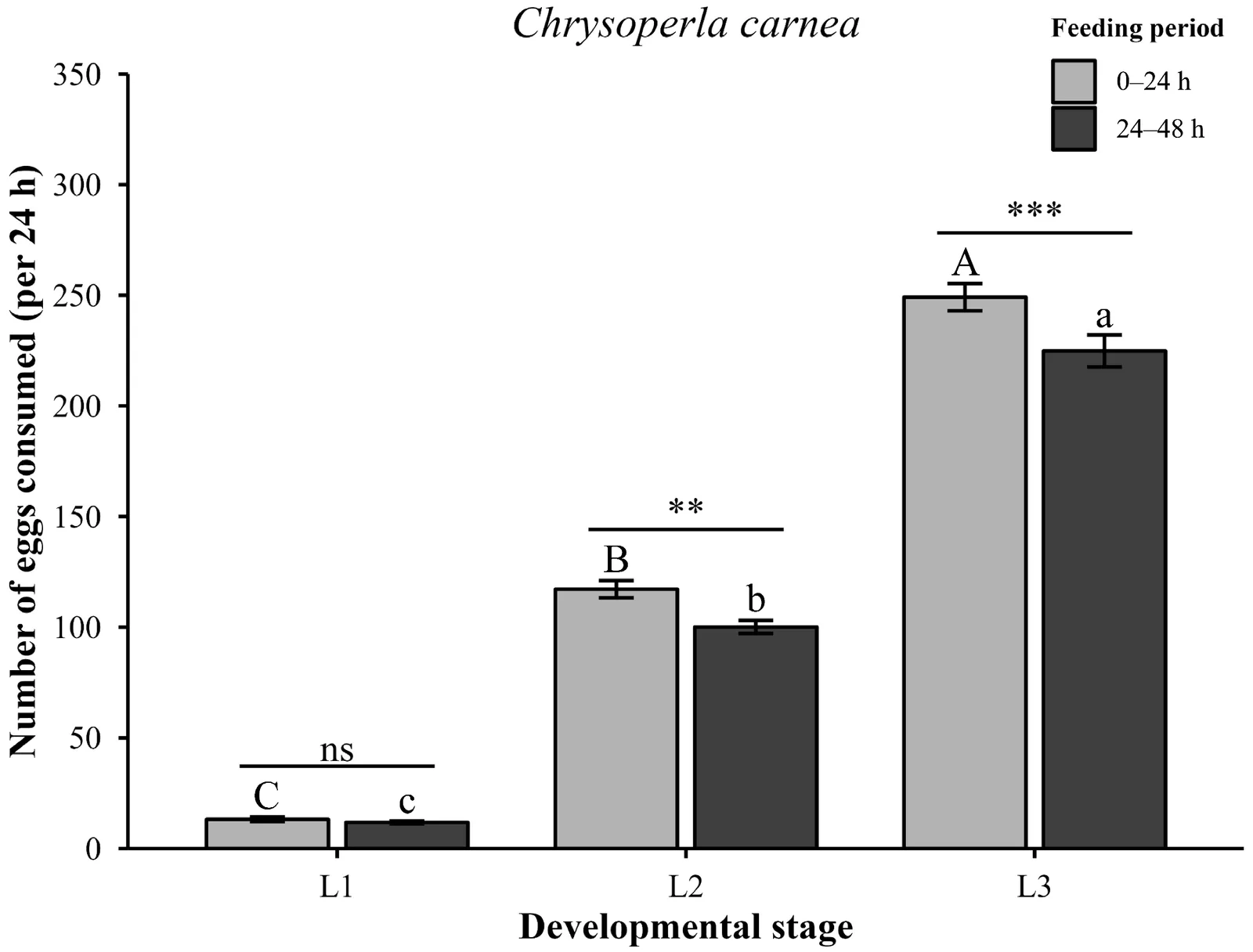
Predation capacity of *Chrysoperla carnea* in the artificial predation assay, presented as the mean (±SE; *n* = 15 replicates) number of *T. absoluta* eggs consumed by *C. carnea* during the L1, L2, and L3 larval instar stages during two sequential 24 h feeding periods respectively spanning hours 0–24 (light-gray bars) and hours 24–48 (dark-gray bars). Statistical test results are presented as detailed in Figure 1’s caption (**, *p* < 0.01, ***, *p* < 0.001, and ns = not significant).

For *C. carnea* larvae, the two-way ANOVA detected a significant interaction between developmental stage and feeding interval (*F*_2,84_ = 3.54, *p* = 0.033), and predation capacity differed significantly among larval instars (*F*_2,84_ = 724.28, *p* < 0.001), while the main effect of feeding interval was not significant (*F*_1,84_ = 0.05, *p* = 0.822) (Figure 2).

Post hoc pairwise tests indicated that predatory voracity increased as the larval stage progressed (L1 < L2 < L3), with L3 larvae demonstrating the highest total predation capacity in the experiment (*p* < 0.001). When evaluating baseline cohort voracity, L2 larvae consumed significantly fewer eggs than L3 larvae but significantly more than L1 larvae (*p* < 0.001), with L1 individuals consistently exhibiting the lowest baseline consumption across both feeding intervals. Regarding satiation over time, egg consumption during hours 24–48 dropped significantly relative to the initial surge observed over hours 0–24 for both the L2 (*p* = 0.007) and L3 (*p* < 0.001) stages. However, no significant temporal shift in feeding rate was observed for L1 larvae (*p* = 0.822), which fed very little overall.

### 3.2. Predation on Tomato Plants

**Figure 3:**
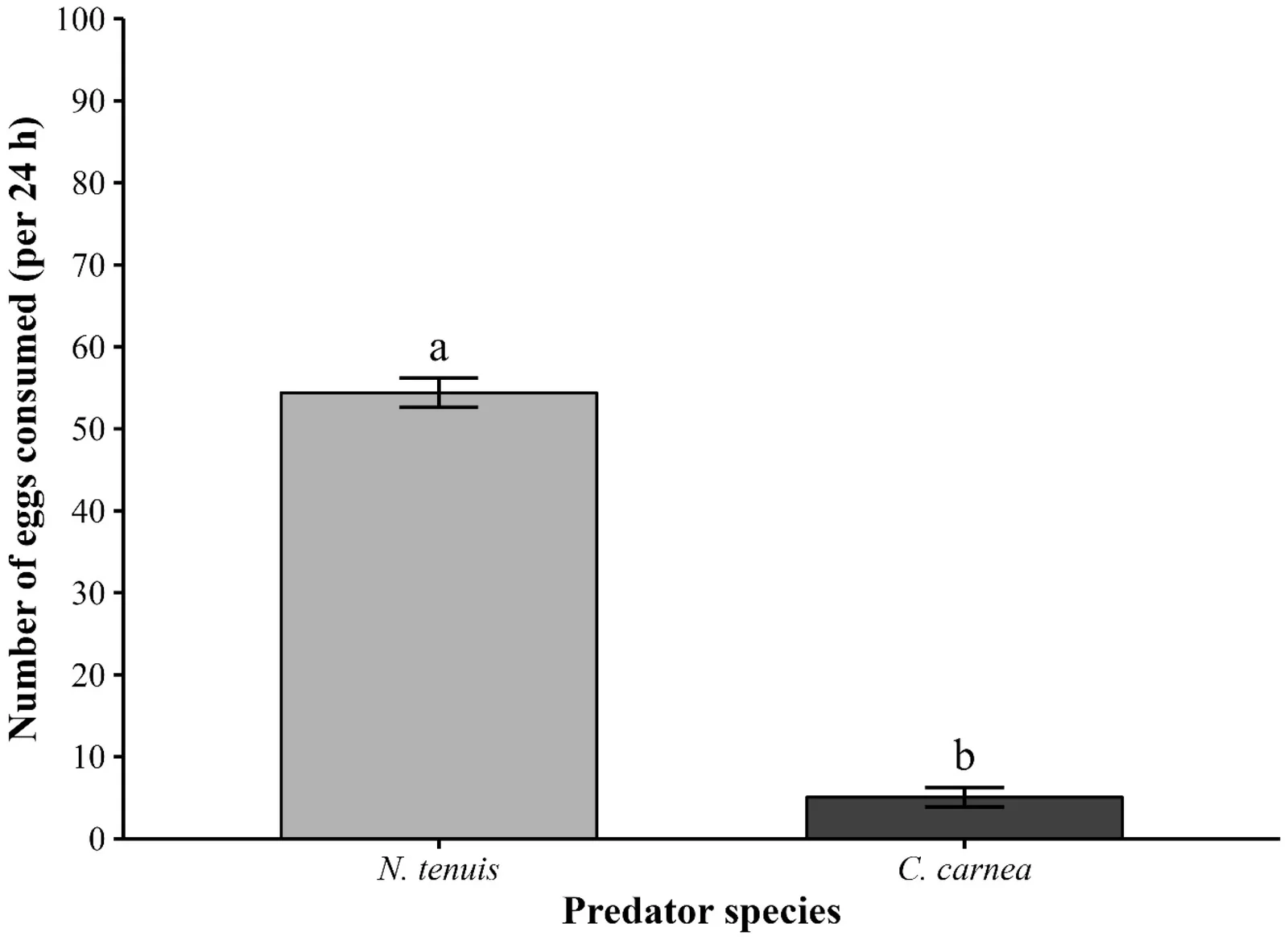
Predation on whole tomato plants. The mean (±SE) *T. absoluta* egg consumption of each predator’s peak predatory cohort (adult females for *N. tenuis* and third-instar larvae for *C. carnea*) was measured on intact tomato plants (*Solanum lycopersicum*) over a 24 h foraging window. The different letters indicate a highly significant difference (*p* < 0.001; independent one-way ANOVA) between the two species.

When individuals at the peak predatory life stage for the two natural enemy species (adult *N. tenuis* females and third-instar *C. carnea* larvae) were evaluated on whole tomato plants, egg consumption differed profoundly between species (one-way ANOVA: *F*_1,28_ = 532.09, *p* < 0.001) (Figure 3).

During the 24 h foraging window, *N. tenuis* females maintained high predatory efficiencies, consuming an average of 54.40 ± 1.78 eggs per plant. In stark contrast, mean consumption by *C. carnea* larvae was recorded at just 5.07 ± 1.19 eggs per plant. This represents a greater-than-tenfold difference in plant-level suppression in favor of *N. tenuis*.

### 3.3. Predatory Capacity of T. absoluta Eggs on Tomato Plants in Semi-Enclosed Cages

**Figure 4:**
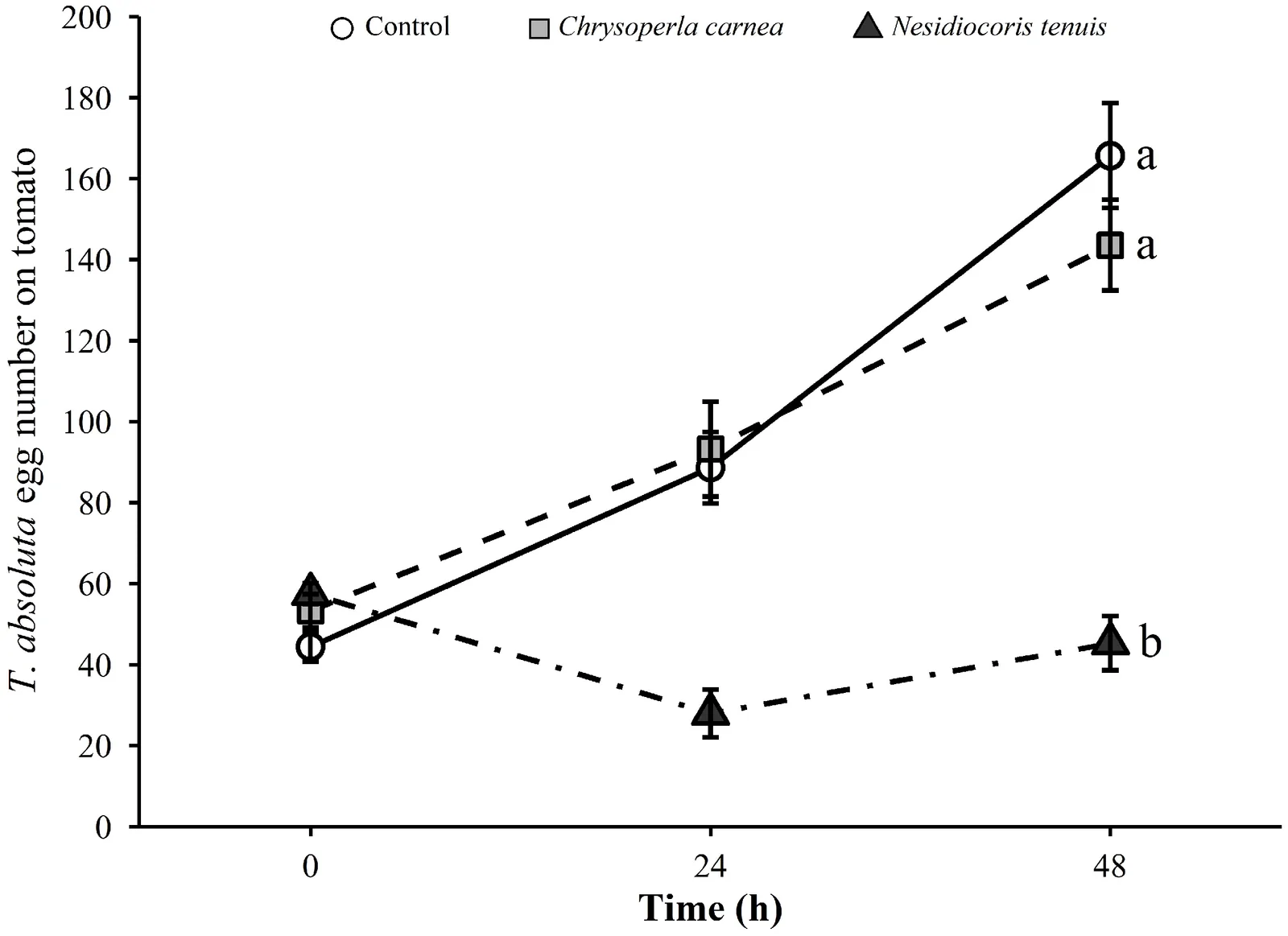
Long-term suppression of *T. absoluta* on tomato plants in semi-enclosed cages under continuous pest oviposition pressure. The absolute numbers of unconsumed *T. absoluta* eggs were measured at 0 h (immediately before predator release), 24 h, and 48 h post-release across two predator treatments, adult *N. tenuis* females and third-instar *C. carnea* larvae, as well as a predator-free control. Different letters associated with the final 48 h timepoints denote statistically significant differences between final egg densities (*p* < 0.05; one-way ANOVA with Tukey’s HSD tests).

To evaluate the predators’ abilities to provide long-term suppression of *T. absoluta* on tomato plants under realistic, multi-generational population dynamics, egg densities were assessed at three sequential time points: immediately prior to predator release (0 h) and at 24 and 48 h post-release (Figure 4). Formal treatment comparisons and hypothesis testing were performed using the absolute values obtained at the final 48 h experimental endpoint.

A one-way ANOVA revealed highly significant differences among the experimental groups in the number of remaining, unconsumed *T. absoluta* eggs (*F*_2,42_ = 36.46, *p* < 0.001) (Figure 4). Post hoc pairwise comparisons using Tukey’s HSD revealed clear differences among the treatment groups.

Adult *N. tenuis* females exhibited robust and highly effective population suppression: from an initial density of 57.5 ± 2.6 eggs per plant at hour 0, they reduced the pest load to a mean of 45.3 ± 6.7 residual eggs per plant at the 48 h timepoint. This final egg density was statistically lower than that observed in all other treatments (*p* < 0.001). Conversely, starting from an initial density of 53.3 ± 4.1 eggs per plant at hour 0, third-instar *C. carnea* larvae failed to provide significant pest suppression, leaving an average of 143.5 ± 11.2 eggs on the foliage 48 h later. In the predator-free control, egg counts increased from an initial baseline density of 44.4 ± 3.7 eggs per plant at hour 0 to 165.7 ± 13.0 eggs per plant at hour 48. Initial egg densities showed no significant differences among treatment groups at hour 0 (*F*_2,42_ = 0.88, *p* = 0.422), and no significant difference in final egg counts was detected between the *C. carnea* treatment and the predator-free control at the 48 h mark (*p* = 0.313).

### 3.4. Predation on Eggplant Plants

**Figure 5:**
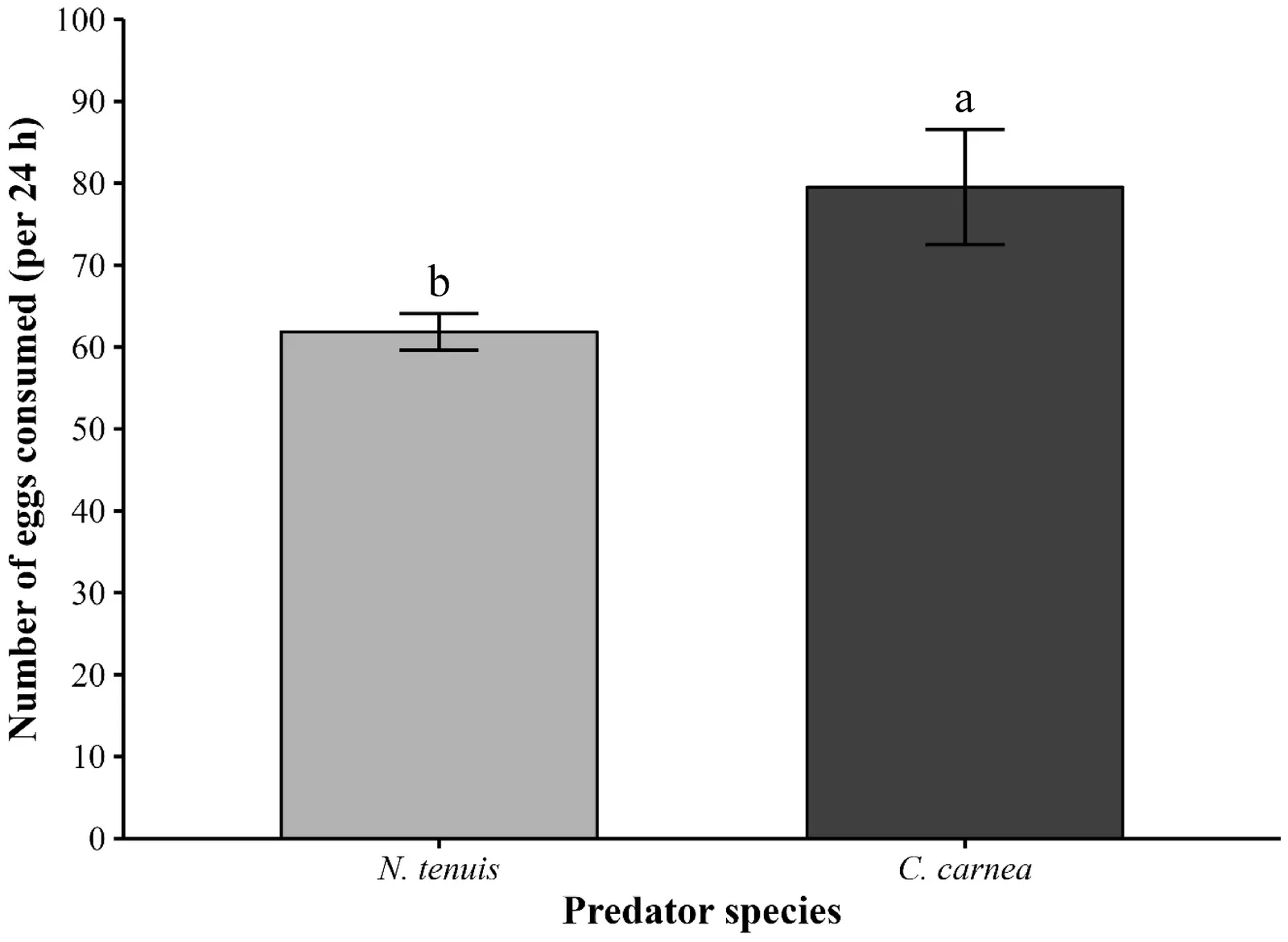
Predatory performances of *N. tenuis* and *C. carnea* on whole eggplant plants over 24 h. The mean (±SE; *n* = 15 replicates) consumption of *T. absoluta* eggs was measured on intact whole plants using both predators’ peak predatory life stages (adult females for *N. tenuis* and third-instar larvae for *C. carnea*). The different lowercase letters above the bars indicate significant differences in predation rates between the two predator species (*p* < 0.05; independent one-way ANOVA).

After the predators were introduced onto the eggplant plant’s surface, the resulting trends in predation efficiency contrasted significantly with those seen in the whole-plant tomato trials (one-way ANOVA: *F*_1,28_ = 5.73, *p* = 0.0237) (Figure 5). Over the 24 h foraging window, third-instar *C. carnea* larvae consistently demonstrated highly active and unimpeded predation, consuming an average of 79.5 ± 7.0 eggs per plant. In comparison, adult *N. tenuis* females consumed a mean of 61.9 ± 2.2 eggs per plant.

### 3.5. Relationships Between Plant Surface Characteristics and Predator Retention

#### 3.5.1. Residency Behavior of *Chrysoperla carnea*

**Figure 6:**
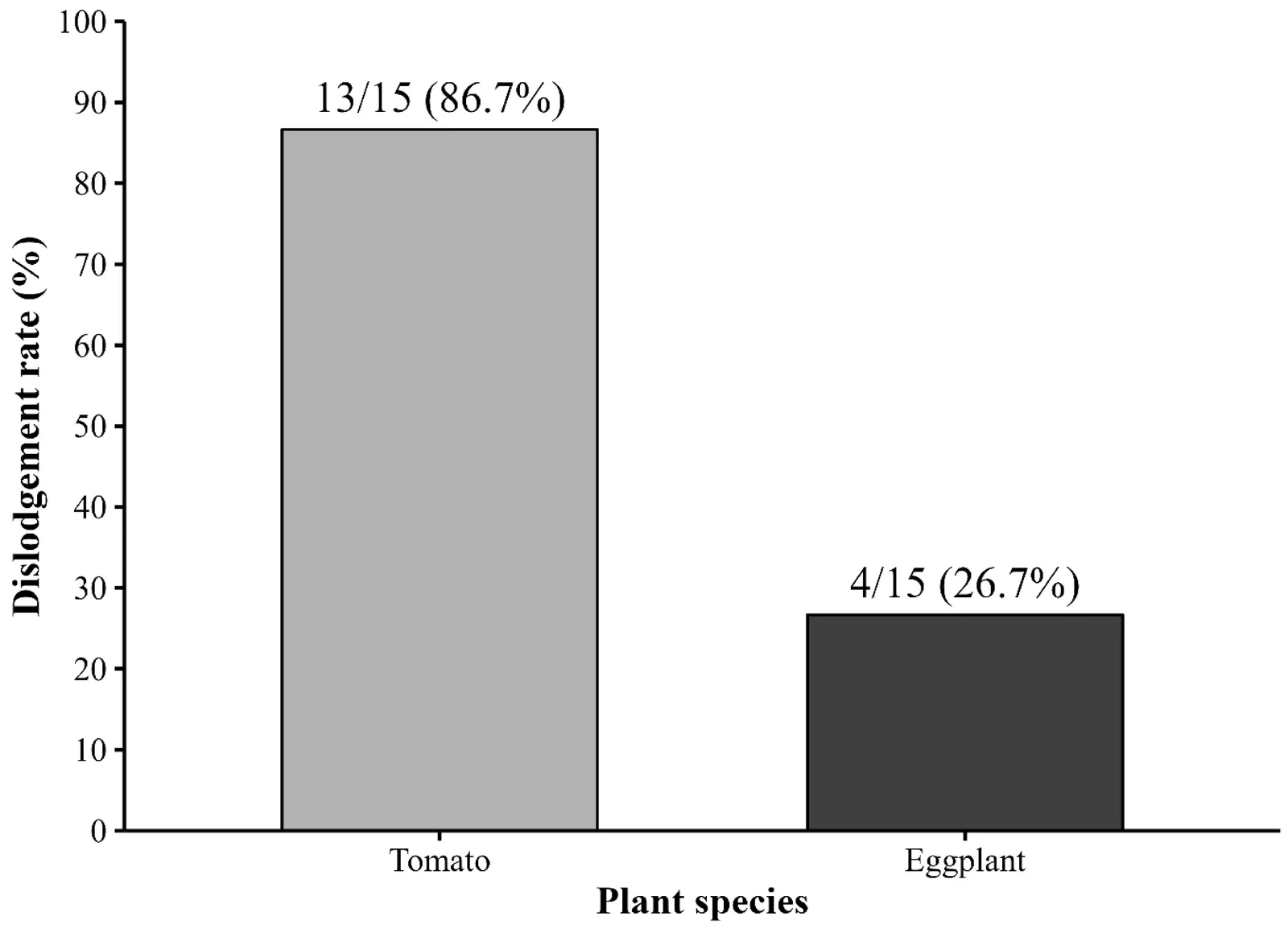
Dislodgement rates (% of individuals no longer on the plant after 24 h) for *C. carnea* on whole tomato (light-gray bar) and eggplant (dark-gray bar) plants. The rates differed significantly between the two host-plant species (*p* < 0.003; Pearson’s chi-square [χ^2^] test of independence).

Directly tracking the status of *C. carnea* third-instar larvae at 24 h post-release revealed a stark, statistically significant divergence in behavioral residency between the two host plant species (Pearson’s chi-square test: *χ*^2^ = 8.69, *df* = 1, *p* = 0.0032). On tomato plants, 13 out of 15 larvae (86.7%) abandoned the canopy and were recovered from the bottom of the cage (Figure 6). Conversely, residency differed starkly on eggplant: only 4 out of 15 larvae (26.7%) abandoned the foliage, while the remaining 73.3% remained on the plant.

#### 3.5.2. Differences in Host Plant Trichome Micro-Morphologies

**Figure 7:**
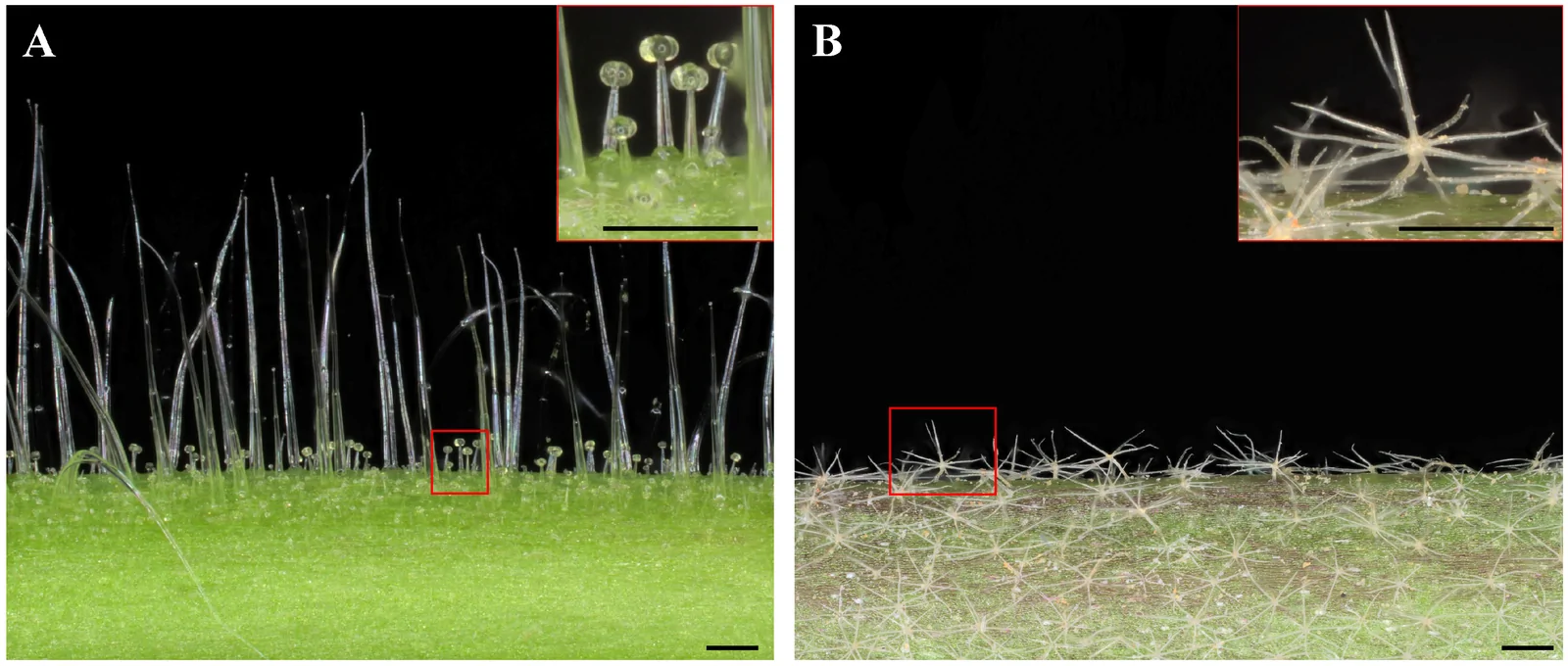
Representative digital micrographs showing the stem surface micro-morphologies and individual trichome details of vegetative (**A**) tomato and (**B**) eggplant plants. Micrographs were taken at 20× (main image) and 100× (inset) magnification in the stem region between 40 and 50 cm above the soil and provide visual validation of the quantitative length differences reported in Table 1. (**A**) Tomato stems feature long, non-glandular trichomes (mean length: 4.14 ± 0.07 mm) interspersed with short glandular trichomes, as shown in the magnified inset (red box). (**B**) Eggplant stems display high densities of short, branched non-glandular trichomes (mean length: 0.48 ± 0.02 mm), with an individual trichome’s morphology clearly shown in the higher magnification inset (red box). All panel scale bars = 0.5 mm.

The stem trichomes of tomato (*Solanum lycopersicum* cv. ‘Seogwang’) and eggplant (*Solanum melongena* cv. ‘Danong’) exhibited distinct morphologies (Figure 7). Tomato stems typically presented two main types of trichomes: long non-glandular trichomes and short glandular trichomes characterized by a large, quadricellular globular head. In contrast, eggplant stem trichomes displayed a porrect-geminate stellate morphology, comprising a multiradiate, non-glandular hair with eight subulate rays and a short central ray.

Quantitative micro-morphological analysis confirmed that the stem surfaces of tomato and eggplant exhibit radically different architectural profiles (Table 1). The mean trichome density on the main stems of eggplant, 489.9/cm^2^, was approximately sixfold that of tomato, 77.4/cm^2^, a significant increase (Welch’s *t*-test: *t*_14.1_ = −9.58, *p* < 0.001). Conversely, the mean length of individual trichomes was nearly ten times greater for tomato, at 4.14 ± 0.07 mm, than for eggplant, at 0.48 ± 0.02 mm, creating a formidable forest of challenging obstacles (Welch’s *t*-test: *t*_34.3_ = 50.15, *p* < 0.001).

## 4. Discussion

This study evaluated the predatory efficacies of two generalist natural enemies, *N. tenuis* and *C. carnea*, against *T. absoluta* across increasing environmental complexities to reveal how plant trichome architecture influences biological control efficiency. In artificial arenas, both predators demonstrated stage-dependent suppression capacities. Prey consumption increased alongside physiological development, peaking in adult females for *N. tenuis* and third-instar larvae for *C. carnea* [25,26,27]. This divergence in peak predatory capacity stems from distinct life-history energy allocation strategies. *N. tenuis* adult females require continuous, protein-rich prey intake to sustain oogenesis and high fecundity. In contrast [12], *C. carnea* is predatory exclusively during its larval stages [15]; its voracious third instar undergoes hyperphagia to store critical nutrients prior to pupation and metamorphosis into a non-predatory adult. For practical application, farmers must align releases with these stage-specific dynamics. Deploying *C. carnea* at the late-larval stage ensures immediate, maximum pest suppression. Conversely, establishing early breeding populations of *N. tenuis* provides sustained, long-term control.

However, when individuals at the best-performing life stages were applied to intact whole plants, the feeding potentials recorded under optimal egg availability failed to predict actual suppression dynamics. On whole tomato plants, *N. tenuis* effectively limited pest numbers, while the predatory efficiency of *C. carnea* was severely suppressed, rendering its control effectiveness statistically indistinguishable from no control at all. Conversely, when evaluated on eggplant plants, *C. carnea* experienced little or no predatory impediment, outperforming *N. tenuis*. This dramatic contrast in performance underscores the necessity of moving past purely descriptive feeding trials to explore the physical and ecological interactions between insect morphology and host crop architecture [17,18]. In open field systems, predator foraging performance is governed by complex tritrophic interactions affected by plant architecture and chemical cues [28]. Herbivore-induced plant volatiles and prey kairomones actively direct predator search behavior, while physical structures like non-glandular trichomes modulate walking speed and leaf residency time [29,30]. These interconnected chemical and physical variables must be considered to understand the ecological complexity of biological control under field conditions.

To resolve these divergent outcomes, our findings can be interpreted through the lenses of habitat complexity theory and trait-mediated predator–plant interactions. Structural complexity within a canopy typically alters predator foraging efficiency by modifying search paths, increasing travel times, and/or reducing prey encounter rates. On tomato plants, long, erect trichomes, averaging 4.14 ± 0.07 mm in length, create a highly complex arrangement of three-dimensional physical barriers that restrict navigation for certain insect groups. This mechanical interference effectively generates an “enemy-free space” for *T. absoluta* eggs on tomato foliage and stems, shielding the pest from large, walking predators [24,31].

Conversely, the micro-morphological profile of eggplant presents a contrasting structural matrix characterized by a significantly higher density of trichomes with a profoundly reduced vertical profile [32,33,34]. Their length and uniform arrangement create a smooth, dense, carpet-like surface that minimizes spatial obstruction. Our behavioral data directly validate these predator–host plant interactions. While most *C. carnea* larvae abandoned the tomato canopy within 24 h, dropping to the cage floor due to a combination of physical dislodgement, locomotive exhaustion, and chemical avoidance triggered by glandular exudates, they maintained steady, continuous residency on the chemically more favorable, physically smoother eggplant foliage.

The divergence in biocontrol efficacy between the two predatory insect species is further illuminated by exploring their contrasting morphologies and locomotive and sensory modalities. *Chrysoperla carnea* larvae are cursorial, sprawling walkers that exhibit continuous, direct tarsal contact with the plant epidermis during navigation, and their foraging strategy is primarily guided by mechanoreceptors located on their legs and maxillary palps, which requires them to sweep across leaf and stem surfaces systematically [30]. When traversing tomato stems, the long, upright trichomes act as potent physical impediments, forcing the larvae to climb over or squeeze between individual stalks. This constant structural friction leads to locomotive frustration, high dislodgement rates, and a complete breakdown of their hunting path. Furthermore, tomato foliage possesses both glandular and non-glandular trichome types, which exert distinct negative impacts on predator mobility [35,36]. While dense non-glandular trichomes form the physical barrier that restricts larval movement, glandular trichomes rupture upon contact and exude sticky, toxic secondary metabolites. These adhesive resins foul the delicate tarsal claws of walking lacewing larvae, pinning them in place or forcing excessive grooming that wastes critical foraging time.

In sharp contrast, *N. tenuis* possesses distinct morphological adaptations that allow it to bypass these physical defenses entirely. As a highly adapted mirid bug, *N. tenuis* possesses elongated, slender legs and specialized pretarsal structures specifically adapted for navigating the sticky, trichome-dense surfaces associated with solanaceous crops [26]. Their long legs elevate the insect’s main body safely above the trichomes, preventing body-to-stem friction. Furthermore, their foraging modality combines visual cues and chemical tracking, allowing them to spot or sense prey from short distances and step precisely between trichome stalks to reach it. Since *N. tenuis* is also phytophagous, it routinely pierces the plant tissue to extract moisture and has developed immunities to the toxic or deterrent chemical exudates that discourage non-adapted predators [13]. This specialized set of features, combining physical elevation, a precise visual–olfactory searching strategy, and chemical tolerance, allows the mirid bug to maintain uninterrupted mobility and high encounter rates across the tomato plant’s complex canopy, where cursorial lacewing larvae fail.

From an integrated pest management (IPM) standpoint, our findings provide clear, practical guidance for selecting biocontrol agents for use in intensive greenhouse production systems. Although *N. tenuis* proved to be the superior predator on tomato, its zoophytophagous nature creates inherent economic risks; at high population densities or when prey becomes scarce, its feeding on the host plant causes necrotic rings on stems and petioles, which can induce severe wilting, reducing yields [13]. Therefore, *N. tenuis* should be employed as the primary biological control agent for *T. absoluta* in tomato greenhouses only during early-to-mid season pest surges, when egg densities are high enough to minimize phytophagy risk, or when closely monitored alongside structural threshold sampling [1,9,11].

Conversely, *C. carnea* cannot be recommended as an effective standalone management tool on long-trichome-bearing tomato cultivars under realistic plant conditions. However, *C. carnea* releases can be highly effective within diversified greenhouse systems that utilize crop rotations or intercropping systems that include smoother, short-trichome-bearing alternate hosts, such as eggplant. On these plants’ surfaces, the lacewing’s exceptional inherent voracity can be fully realized, providing a safer biocontrol alternative that carries zero risk of crop injury.

As this study focused on short-term predation responses on young vegetative plants, several ecological parameters require further investigation. Future research should include field-scale, multi-generational trials to assess both the predator and prey insects’ long-term survival, reproduction, and population persistence under fluctuating environmental conditions. Furthermore, investigating the spatial and temporal distribution of predators in commercial fields will be necessary to optimize mass release strategies. Since trichome density, length, and chemical exudate profiles change drastically during plant ontogeny, subsequent work must evaluate how transitioning from the vegetative to mature flowering/fruiting stages, as well as intermediate crops like pepper, influence predator efficiency. Finally, evaluating multi-predator combinations is highly warranted to determine if simultaneous releases yield positive foraging synergies or cause negative interspecific interference.

## 5. Conclusions

This study demonstrates that host crop surface architecture, rather than predatory voracity alone, dictates the biological control efficacies of *N. tenuis* and *C. carnea* against *T. absoluta*. Given these findings, *N. tenuis* remains preferable for current tomato cultivars, whereas *C. carnea* should be prioritized for short-trichome crops like eggplant or intercropping systems. Ultimately, these results highlight that effective biological control hinges on strategically aligning natural enemy selection with the physical and chemical surface traits of target crops.

## Figures and Tables

**Table 1:** Micro-morphological characteristics of tomato and eggplant stem surfaces. Measurements (mean ± SE) are derived from 15 independent biological replicates (*n* = 15 plants per species).

Parameter	Tomato	Eggplant	*p*-Value
Trichome density (no./cm^2^)	77.4 ± 2.2	489.9 ± 43.0	*p* < 0.001
Trichome length (mm)	4.14 ± 0.07	0.48 ± 0.02	*p* < 0.001

Note: Statistically significant differences between the two host plant species were assessed for each parameter using an independent-sample Welch’s *t*-test due to unequal variances, with *p*-values shown in the right column.

## Data Availability

The original contributions presented in this study are included in the article. Further inquiries can be directed to the corresponding authors.
